# Plant Conservation Genetics Advocates for a Green List

**DOI:** 10.1002/ece3.74628

**Published:** 2026-10-30

**Authors:** Alison Gonçalves Nazareno, Loreta Brandão de Freitas, Christine E. Edwards

**Affiliations:** ^1^ Department of Genetics, Ecology, and Evolution Federal University of Minas Gerais Belo Horizonte MG Brazil; ^2^ Departamento de Genética, Instituto de Biociências Universidade Federal do Rio Grande do Sul Porto Alegre RS Brazil; ^3^ Missouri Botanical Garden St. Louis Missouri USA

**Keywords:** effective population size, genome‐wide studies, plant genetics resources, population genetics

## Abstract

Our definition of a green list is rooted in the art of safeguarding plant genetic resources and relies on the ability to compile all the tools and information on conservation plans and management activities with the goal of de‐listing any taxon of special concern from the red lists.

The Special Issue “Practical Trends in Plant Conservation Genetics” presents manuscripts focused on the conservation of genetic diversity for plant species of special concern. The manuscripts featured in this special issue are summarized in Table 1. We refer to this list as the “Green List”—which encompasses the goals and primary results of the studies, notes on management and conservation recommendations, and additional information regarding plant species of special concern. Because this list summarizes conservation recommendations for imperiled plants, we feel that such a list, encompassing research on threatened plant species, would be useful for coordinating conservation efforts going forward. Of the topics published in the special issue, we would like to mention the following as likely to interest to the general reader: population genomics (Abate et al. 2025; Baker, Cottrell, Ennos, et al. 2025; Brunton et al. 2025; Chen et al. 2026; Neel et al. 2025; Pollegioni et al. 2025; Xie et al. 2026), quantitative genetics (Baker, Cottrell, Ennos, Perry, et al. 2025), transcriptomics (Yang et al. 2024), biogeography (Aihara et al. 2025), phylogenomics (Li et al. 2026), and conservation genomics (Eastman‐Densem et al. 2025). Below, we highlight some important practical trends in conservation genetics that should be considered for future studies.

**TABLE 1:** The Green list of plant species receiving special attention. The table depicts the goals and results related to the management and conservation of plant genetic resources, including genetics, biological and ecological aspects, recovery, and funding information, as included in the Special Issue ´Practical Trends in Plant Conservation Genetics´.

Authors	Species	Goals	Main results	Research funds
Brunton, Page, and Lee	*Santalum* L. spp.	Identifying the limits and evolutionary biology of multiple lineages to enhance conservation activities for the sandalwood in Australia	The key message is that conservation should not rely solely on the species as the unit of management. The results demonstrate that different populations may represent genetically distinct lineages, each with unique evolutionary histories and potential local adaptations. Therefore, conservation strategies should integrate genomics, reproductive biology, geographic distribution, biogeographic history, and ecology.	Australian Centre for International Agricultural Research (ACIAR)
Li, Qiu, Li, Xu, Yu, and Ju	*Aletris* L. spp.	Characterizing plastid genomes, contributing to improve taxonomy and identifying the evolutionary relationships among species leading to a better understanding of local biodiversity	The authors identified a new, potentially vulnerable taxonomic and evolutionary unit and developed genomic tools for its conservation. This article's contribution to the management and conservation of plant genetic resources is indirect yet highly significant. It demonstrates how taxonomy, genetics, ecology, and conservation are interconnected.	Science and Technology Major Project of Xizang, Special Investigation and Monitoring of the Yarlung Zangbo Grand Canyon National Nature Reserve in Xizang, and Second Tibetan Plateau Scientific Expedition and Research program
Xie, Jin, Yang, Lu, Zhang, Zhang, and Jiang	*Primula wilsonii* Dunn	Combining population genomics, demographic history, and species distribution modeling to propose management units and conservation strategies, thereby filling a knowledge gap regarding genetic vulnerability and providing a scientific basis for the long‐term conservation of a species with extremely small populations.	The results indicated that complex topography and paleoclimatic oscillations have shaped a hierarchical genetic structure with deeply isolated lineages within the species. Therefore, the authors recommend delineating at least three independent management units, consistent with patterns of genetic structure, differentiation, and demographic history. These findings highlight the importance of integrating genetic and environmental evidence to conserve species with small populations.	National Natural Science Foundation of China and Natural Science Foundation of Anhui Province
Chen, Sun, Wu, and Huang	*Holcoglossum pumilum* Hayata	Using genomic data to guide conservation policies and strategies, particularly because many wild orchids lack *ex situ* gene banks and are subject to collection, habitat loss, and climate change.	This article is directly applicable to genetic conservation. Its main contribution is demonstrating that a species classified as “Least Concern” can exhibit a very small contemporary effective population size—and thus a genetic vulnerability—that is not captured by conventional conservation status. For management purposes, certain populations constitute priority genetic reservoirs due to their greater diversity and adaptive potential. At the same time, the absence of strong genetic structuring indicates that these populations can provide material for reintroduction or genetic rescue, provided that multiple sources are used, environmental matching is ensured, and subsequent genomic monitoring is conducted.	Biodiversity Research Center, Academia Sinica and the National Science and Technology Council
Aihara, Cheng, Chen, Wang, Uchiyama, Takahashi, Suyama, and Tsumura	*Chamaecyparis obtusa* (Siebold & Zucc.) Endl.	The study employs a large‐scale genomic approach to reconstruct the biogeographic history of a relict species of significant economic importance and, based on current genetic structure, proposes strategies for the conservation of natural populations and the management of individual transfers to maintain the genetic diversity of these populations.	The current classification used for the transfer of the species' material—based primarily on climatic differences—does not adequately correspond to the genetic units identified in the study. The authors consider these lineages conservation priorities because they harbor genetic variants resulting from long histories of isolation and adaptation. Furthermore, conservation efforts for the species should not focus solely on large or economically important populations. Marginal populations may possess high genetic value, making it necessary to consider local adaptation when moving propagation material. The authors emphasize that highly divergent populations require special conservation attention, recommending that seedlings not be transferred between distinct genetic clusters.	Japan Society for the Promotion of Science
Pollegioni, Marchesini, Gaudet, Chiocchini, Monti, Leonardi, Cherubini, and Mattioni	*Alnus cordata* (Loisel.) Duby	The study is directly related to the management and conservation of forest genetic resources. It combines population genetics, hybridization, climate suitability modeling, and spatial prioritization to determine which populations should be conserved in situ and which gaps warrant *ex situ* strategies. The main aim was to assess the genetic diversity of an endemic tree species and use this information to develop a conservation strategy in the face of climate change.	The authors concluded that current in situ conservation may not be sufficient to preserve genetic resources in the long term, as some genetically important populations could find themselves in climatically unsuitable areas in the future.	National Biodiversity Future Center; European Union—NextGenerationEU; and CNR USEit: Utilizzo Sinergie operative per la gestione integrata specie aliene Invasive in ITalia
Yang, Li, Xie, Yuan, and Guo	*Litsea coreana* Levl. var. *sinensis*	The primary focus is the characterization and utilization of the genetic variability of a plant resource of economic interest, particularly for germplasm selection, breeding, and the production of tea with higher flavonoid content. To this end, the authors characterized the existing genetic diversity and identified variants of interest that can be preserved and used in tea germplasm improvement.	The authors concluded that the observed phenotypic and chemical diversity is strongly related to differences between habitats. This means that, to adequately conserve the genetic potential for flavonoid production, preserving genotypes is not enough; it is also necessary to consider the environments in which these genotypes evolved and are cultivated.	National Natural Science Foundation of China
Eastman‐Densem, Glenny, Heenan, Wold, and Pelser	*Gentianella calcis* Glenny & Molloy	The study integrates conservation genomics, population structure, connectivity, intraspecific differentiation, and ecological characteristics to define conservation units and guide population prioritization.	The results show that a species endemic to calcareous habitats and threatened with extinction exhibits high genetic differentiation among populations, low connectivity, and possible local adaptation, indicating that these populations constitute important and irreplaceable components of the genetic diversity in this species. The authors recommend conserving all remaining populations, ensuring the preservation of at least one population from each of the four subspecies. Protecting calcareous habitats is also essential.	Miss E. Hellaby indigenous grasslands trust and Ministry of Business Innovation and Employment for the Strategic Science Investment Fund
Baker, Cottrell, Ennos, Perry, Green, and Cavers	*Juniperus communis* L.	The study evaluated a native tree species in a common garden experiment to determine how much of the phenotypic variation is explained by differences among regions, populations, and families, and whether there is evidence of local adaptation.	The study demonstrates that the remaining populations exhibit phenotypic differentiation consistent with genetic isolation and possible local adaptation, reinforcing the need to conserve multiple populations and their natural regeneration processes; this entails promoting natural regeneration and avoiding indiscriminate plantings that could alter the local genetic structure. The study provides a baseline to guide the conservation of the species, its adaptive potential, particularly in light of habitat fragmentation, low regeneration rates, and its threatened status.	The Scottish Forestry Trust, Forest Research, The Botanist Foundation, The Woodland Trust, and the UK Centre for Ecology & Hydrology
Neel, Marsden, and Engelhardt	*Vallisneria americana* Michx.	The study investigates the genetic structure of an aquatic macrophyte that is fundamental to riverine ecosystems, as it is a submerged plant and a foundational species in these environments. Its presence contributes to the structuring of the aquatic habitat and provides ecological benefits to other organisms.	The study is particularly relevant to genetic conservation and restoration, as it demonstrates that hydrological connectivity does not necessarily guarantee genetic connectivity and that restoration strategies must take local genetic structure into account. The authors emphasize that individuals of the species found along the river should not be viewed as belonging to a single gene pool—a fact with direct implications for restoration: the origin of the material used must consider the individual's location as well as the genetic diversity of both the source and destination sites.	U.S. Environmental Protection Agency (EPA) and Science To Achieve Results (STAR) Fellowship
Abate, Degu, Kalousová, and Abebe	*Afrocarpus gracilior* (Pilg.) C. N. Page	The authors sought to evaluate whether forest fragmentation is associated with genetic erosion and inbreeding depression in a wind‐pollinated, dioecious conifer, and whether these genetic changes are reflected in progeny performance. Specifically, they assessed differences between small/isolated populations and larger populations regarding seed quality, germination, survival, and early seedling vigor, relating these traits to genetic diversity.	Low diversity and strong genetic drift indicate genetic erosion associated with fragmentation, with greater loss of diversity in small, isolated populations. All populations exhibited private alleles; therefore, even small ones contribute uniquely to the species' gene pool and should not simply be disregarded for conservation purposes. The primary recommendation is to prioritize the conservation of larger and more genetically diverse populations, particularly natural forest remnants and sacred sites that harbor greater genetic diversity. These populations should be identified and, ideally, designated as priority seed sources for restoration.	Hawassa University, Czech Ministry of Foreign Affairs, and Internal Grant Agency of the Czech University of Life Sciences (CZU) Prague
Baker, Cottrell, Ennos, Perry, A'Hara, Green, and Cavers	*Juniperus communis* L.	The study used molecular markers to assess genetic diversity, structure, and population dynamics, guiding the prioritization of populations, conservation monitoring, and potential genetic rescue actions for a species considered a keystone for the communities in which it occurs, providing habitats for lichens and bryophytes and abundant seasonal forage for animals.	The results show that the studied populations are not completely isolated, yet there are substantial barriers to gene flow, particularly among the three main population centers. The regions also exhibited distinct patterns of genetic diversity and population structure, indicating that internal gene flow varies among them. The authors suggest a “dynamic conservation” approach, aimed at maintaining or increasing genetic diversity through natural processes—primarily gene flow, natural selection, sufficiently large populations, and natural regeneration.	newLEAF, Forest Research, The Botanist Foundation, The Scottish Forestry Trust, UK Centre for Ecology and Hydrology, and The Woodland Trust

## 1 Data Filtering

While all studies in the special issue advocate for conserving plant genetic resources, Eastman‐Densem et al. (2025) and Chen et al. (2026) highlight the importance of careful consideration of filtering parameters for genomic data before employing it for downstream analyses. In fact, Chen et al. (2026) reported that contemporary effective population size—a paramount parameter in conservation plans—varied more than fourfold when missing data ranged from 20% to 50%, with datasets with greater values of missing data and more loci corresponding to smaller estimates of effective population size and those with lower numbers of loci and proportions of missing data showing greater effective population size. Because the percentage of missing data is study‐dependent (Nazareno and Knowles 2021), is usually positively associated with the number of loci, and both vary based on filtering criteria, choosing an appropriate dataset should be based on the variance of the population genetic parameters.

Here, we would like to emphasize that data filtering procedures, such as removing non‐neutral loci from raw data (i.e., loci under selection, mitochondrial and plastid loci, those not conforming to Hardy–Weinberg Equilibrium, and those showing strong Linkage Disequilibrium), are also vital trends that should be followed in conservation genetics, as observed, for example, in Aihara et al. (2025), Baker, Cottrell, Ennos, et al. (2025), Eastman‐Densem et al. (2025), and Xie et al. (2026). Furthermore, an appropriate minor allele frequency (MAF) threshold must be considered, as it is sample‐dependent.

## 2 Calculating the Time to Minor Allele Fixation

Following Crow and Kimura (1970), we would like to stress, as another practical trend, that both genome‐wide studies and those that have used very few genomic regions (e.g., population genetics studies employing co‐dominant bi‐allelic molecular markers) should calculate and report an estimate of the mean number of generations (*t*) until the average MAF is fixed by genetic drift. This estimate should be refined further by considering a metapopulation dynamic in which the strength of each of the microevolutionary forces, not only genetic drift, acting among alleles, is equal and related to the size of the population (e.g., mutation events are prone to occur and be inherited more frequently in larger than smaller populations). Hence, *t* can be estimated, for instance, from the original equation of Crow and Kimura (1970), replacing *p* (allele frequency) with the average minor allele frequency.

$$\begin{array}{c} t=\frac{-4\;\mathrm{Ne}\;(\mathrm{MAF})\ln\mathrm{MAF}}{1-\mathrm{MAF}} \end{array}$$

Even if the sample and reproductive population sizes are known, because MAF is directly related to Ne, estimating *t* would be helpful for understanding the trajectories of populations, especially for small populations of threatened plant species on the verge of extinction.

Deriving data from Chen et al. (2026), for example, some populations of the imperiled orchid *Holcoglossum pulmillum* may be lost in two generations due to extremely low levels of genetic diversity, given its contemporary effective size of 12 and a MAF as low as 7.8 × 10^−3^, assuming it is a diploid plant species with a population size of 64 and an allele count of one in 128 as a mean. Keeping the number of orthologous loci and the contemporary effective population size equal to 12 (Chen et al. 2026), but changing the sampling approach—for example, sampling to assess the minimum distance between seed‐trees to collect seeds to recover plant populations; estimating the net migration amount among populations to design and implement ecological corridors—producing a MAF value of 10%, for instance, would result in a *t* value of ca. 12 generations, indicating that the mean value of the number of generations must be treated with caution, as MAF depends on sampling approach. Notice that irrespective of the MAF threshold applied to filter the data, the average value of MAF can be obtained directly from the output file of the Populations program in STACKS (Catchen et al. 2013), for example, and this value must be used to calculate *t*.

## 3 Other Experimental Design and Analytical Considerations

That aside, we would like to argue that population genetics studies that are based on whole genome or reduced‐representation sequencing methods (e.g., RNAseq, GBS, ddRADseq, MIG‐seq, DArTseq) should (i) include biological and technical replicates to identify, correct, and reduce potential biases in genetic parameters, and (ii) employ subsequent analyses that do not violate the assumptions of the Neutral Theory; for example, use the conStruct program (Bradburd et al. 2018) instead of Admixture (Alexander et al. 2009)/Structure (Pritchard et al. 2000) when an Isolation by Distance pattern is observed (but see Aihara et al. 2025, Baker, Cottrell, Ennos, Perry, et al. (2025), Eastman‐Densem et al. 2025), or use InStruct (Gao et al. 2007) when a population is highly inbreeding.

Last but not least, we would like to congratulate and recognize all the authors for their efforts to conserve and safeguard plant genetic resources, and to help us envision the Green List—an idea still in its infancy.

## Author Contributions

**Alison Gonçalves Nazareno:** conceptualization (lead), writing – original draft (lead). **Loreta Brandão de Freitas:** writing – review and editing (equal). **Christine E. Edwards:** writing – review and editing (equal).

## Funding

The authors have nothing to report.

## Ethics Statement

Ethical approval was not required for this study because it was based exclusively on previously published literature.

## Conflicts of Interest

The authors declare no conflicts of interest.

## Data Availability

No new data were generated or analyzed in this study.
